# Prediction of anterior chamber volume after implantation of posterior chamber phakic intraocular lens

**DOI:** 10.1371/journal.pone.0242434

**Published:** 2020-11-16

**Authors:** Yuki Takagi, Takashi Kojima, Tomoya Nishida, Tomoaki Nakamura, Kazuo Ichikawa

**Affiliations:** 1 Department of Ophthalmology, Iida Municipal Hospital, Nagano, Japan; 2 Nagoya Eye Clinic, Nagoya, Japan; 3 Department of Ophthalmology, Keio University School of Medicine, Tokyo, Japan; 4 Chukyo Eye Clinic, Nagoya, Japan; University of Warmia, POLAND

## Abstract

**Purpose:**

To predict the anterior chamber volume (ACV) after implantable collamer lens (ICL) implantation based on ICL size and parameters of anterior segment optical coherence tomography (AS-OCT).

**Design:**

Retrospective study.

**Methods:**

This study included 222 eyes of 222 patients who underwent ICL implantation at Nagoya Eye Clinic. The patients were divided into two groups: prediction group, for creating the prediction equation (148 eyes, mean age: 32.11 ± 8.04 years), and verification group, for verifying the equation (74 eyes, mean age: 33.03 ± 6.74 years). The angle opening distance (AOD), anterior chamber width (ACW), ACV, anterior chamber depth, lens vault, angle-to-angle distance, angle recess area, and trabecular iris space area were calculated using AS-OCT. A stepwise multiple regression analysis was performed. After the creation of the prediction equation, its accuracy was verified in the verification group.

**Results:**

The ACV, AOD750, ACW, and ICL size were selected as explanatory variables to predict postoperative ACV. Mean predicted (114.2 ± 21.83 mm^3^) and actual postoperative ACVs (116.1 ± 25.41 mm^3^) were not significantly different (P = 0.269); absolute error was 10.59 ± 9.13 mm^3^. In addition, there was high correlation between actual and predictive ACV (adjusted R^2^ = 0.6996, p < 0.0001). Bland-Altman plot revealed that there was no addition or proportional error between predicted and actual postoperative ACV.

**Conclusion:**

Postoperative ACV was accurately predicted using AS-OCT parameters and ICL size. This prediction equation may be useful for making decisions regarding ICL size.

## Introduction

Implantable collamer lens (ICL; STAAR Surgical, Nidau, Switzerland) was initially used for refractive correction in patients with high myopia. Recently, it was reported to be safe and effective even for the treatment of mild and moderate myopia [1]. ICL implantation has more advantages than do laser kerato-refractive surgeries such as laser in situ keratomileusis (LASIK); the former is associated with no cornea-related complications, such as corneal ectasia and chronic dry eye disease [2]. Cataract and pupillary block are known as serious complications after ICL implantation. A challenging issue in ICL implantation surgery is the determination of the ICL size. The conventional ICL sizing method specified by the manufacturer used horizontal corneal diameter and anterior chamber depth, but the angle condition was not considered. Several studies have shown that the ICL size can be accurately determined to ensure the optimum height for the postoperative vault (distance between ICL and crystalline lens) [3–5]. However, there are various postoperative angle conditions even among similar vaults. Therefore, we suggested that the ICL size should be determined so that both the vault and the angle condition are optimal.

In patients with chronic angle closure, angle opening distance (AOD), anterior chamber volume (ACV), and trabecular iris space area (TISA) were useful for evaluating the risk of angle closure. Among those parameters, ACV was reported to be the most clinically important parameter to assess the status of angle closure [6, 7]. It was reported that anterior chamber distance (ACD) and angle change after ICL implantation [8, 9]. A previous report showed that intraocular pressure (IOP) was elevated by angle closure after ICL implantation [10]. ACV measured by anterior segment optical coherence tomography (AS-OCT) was useful for observing the change of anterior segment and angle structure after ICL implantation [11]. Based on these studies, we presumed that ACV would be useful for predicting the anterior chamber angle after ICL implantation.

Using Scheimpflug-based corneal tomography, a previous study showed that ACV significantly decreased after ICL implantation and that changes in ACV were correlated with preoperative ACV, preoperative ACD, preoperative horizontal anterior chamber area (ACA), and ICL vault [11]. In addition, the same study reported that postoperative ACV was predicted by four parameters, including preoperative ACV, preoperative horizontal ACA, preoperative ACD, and postoperative vault. However, there are no reports evaluating the prediction of postoperative ACV using only preoperative parameters. Given this gap in the literature, we examined whether the size of the implanted ICL and preoperative AS-OCT parameters may be used to predict postoperative ACV. Moreover, we generated a prediction equation for ACV and verified it using a different group of patients.

## Materials and methods

### Patients & study design

We retrospectively prescreened consecutive patients who underwent ICL implantation at the Nagoya Eye Clinic from May 2016 to May 2018. Among them, we included 222 eyes of 222 patients (mean age; 32.4 ± 7.6 years, 100 men and 122 women) who finished a 3-month follow up after surgery and had no complications both during and after the surgery. Patients who had a history of systemic disease or ocular disease other than refractive error were excluded. This retrospective study was approved by the institutional review board (Nagoya Eye Clinic, #2019–33), and the study followed the Declaration of Helsinki. An opt-out method was approved by the institutional review board as an alternative to obtaining informed consent.

### ICL implantation surgery

The VICM5 or TVICM5 ICL model was used in all patients. The refractive power of ICLs was decided using the online software provided by the manufacturer. The ICL size was determined using AS-OCT according to a previously reported method [4]. ICL implantation was performed as described in a previous report [12]. Briefly, after the topical administration of anesthesia using anesthetic eye drop, a 3.2-mm temporal corneal incision was made. After the anterior chamber was filled with viscoelastic material, the ICL was injected into the anterior chamber using an injector, and subsequently, four haptics were placed under the iris using an ICL manipulator. The viscoelastic material was removed using an irrigation and aspiration system. Finally, 0.1 mL of acetylcholine (OVISOT, Daiichi Sankyo, Tokyo, Japan) was injected into the anterior chamber for pupil miosis.

The patients were randomly divided into the “prediction” group for generating the prediction equation and the “verification” group for verifying the equation. Randomization was performed using a random numbers table in Excel (Microsoft, Redmond, Washington). Patient demographic information of both groups is shown in Table 1. Initially, we analyzed the single correlation between the postoperative ACV and preoperative parameters in the prediction group. Subsequently, a stepwise multiple regression was analyzed with ACV after ICL implantation as a dependent variable, and preoperative AS-OCT parameters and ICL size as independent variables, and the ACV prediction equation was generated. Finally, the accuracy of the prediction equation was evaluated in the verification group.

**Table 1:** Patient demographic information.

	Prediction group	Verification group
No. of patients	148	74
Sex (male, female)	64, 84	36, 38
Age (years)	32.11 ± 8.04	33.03 ± 6.74
Manifest refractive sphere (D)	−7.34 ± 2.44	−7.98 ± 2.48
Preoperative IOP (mmHg)	13.7 ± 3.00	14.1 ± 2.66

IOP, intraocular pressure; D, diopter.

Values were presented as mean ± standard deviation

The sex was verified through health insurance cards.

### Anterior segment OCT measurement

AS-OCT CASIA2 (TOMEY, Nagoya, Japan) was used in this study. All parameters were calculated using the internal analysis software of the AS-OCT. AS-OCT measurement was performed with illuminance settings of 150 lux. Although both eyes were examined using AS-OCT, the data from the right eye were used for analyses. The representative angle parameters, including AOD from the horizontal quadrants, were used for analyses. Postoperative AS-OCT parameters were measured at 3 months after the surgery. All AS-OCT measurements were carried out by well-trained certified orthoptists. The AS-OCT parameters mentioned in this paper are provided in the S1 Fig.

### Statistical analysis

The data were analyzed using SPSS (SPSS, Chicago, IL) software, and the statistical significance level was set at <5% (P < 0.05). Single correlation analysis was performed using the Pearson correlation coefficient. To create the prediction equation of postoperative ACV, stepwise multiple regression analysis was performed. The accuracy of the prediction equation was assessed in the verification group using Bland-Altman plots. All relevant data are included in this manuscript and its Supporting Information files.

## Results

### Single correlation of postoperative ACV with preoperative AS-OCT parameters/ICL size

The results of the single correlation are demonstrated in Table 2. Postoperative ACV showed significant positive linear correlations with preoperative ACV (r = 0.762, P < 0.001), anterior chamber width (ACW) (r = 0.564, P < 0.001), and AOD750 (r = 0.465, P < 0.001). However, there was no correlation between postoperative ACV and ICL size (P = 0.202). In addition, the mean postoperative vault was 0.63 ± 0.26 mm (min: 0.039 mm; max: 1.4 mm).

**Table 2:** AS-OCT parameters and results of single linear regression analysis with postoperative ACV.

	Mean ± SD	Range (Min, Max)	Adjusted R^2^	P
ACV (mm^3^)	190.03 ± 27.40	131.53, 251.23	0.762	<0.001
ICL size (mm)	12.80 ± 0.34	12.1, 13.2	0.069	0.202
ACW (mm)	11.84 ± 0.41	10.82, 12.95	0.564	<0.001
ACD (mm)	3.312 ± 0.25	2.77, 3.98	0.652	<0.001
LV (mm)	−0.15 ± 0.19	-0.53, 0.27	−0.378	<0.001
AOD250 (mm)	0.40 ± 0.16	0.081, 0.85	0.314	<0.001
AOD500 (mm)	0.62 ± 0.21	0.22, 1.18	0.413	<0.001
AOD750 (mm)	0.87 ± 0.26	0.35, 1.6	0.465	<0.001
ARA250 (mm^2^)	0.09 ± 0.051	0.003, 0.28	0.243	0.001
ARA500 (mm^2^)	0.23 ± 0.094	0.036, 0.53	0.314	<0.001
ARA750 (mm^2^)	0.42 ± 0.15	0.14, 0.86	0.377	<0.001
TISA250 (mm^2^)	0.08 ± 0.035	0.003, 0.19	0.285	<0.001
TISA500 (mm^2^)	0.21 ± 0.80	0.036, 0.43	0.340	<0.001
TISA750 (mm^2^)	0.40 ± 0.13	0.14, 0.76	0.396	<0.001
TIA250 (deg)	54.97 ± 14.30	17.9, 119.3	0.353	<0.001
TIA500 (deg)	49.81 ± 11.48	23.9, 98.1	0.472	<0.001
TIA750 (deg)	48.05 ± 9.44	26.1, 87.1	0.520	<0.001
I-Curv (mm)	-0.01 ± 0.12	-0.27, 0.21	-0.371	<0.001

Values are presented as mean ± standard deviation.

ACV, anterior chamber volume; ACW, anterior chamber width; ACD, anterior chamber depth; LV, lens vault; AOD, angle open distance; ARA, angle recess area; TISA, trabecular iris space area: TIA, trabecular iris angle

### Stepwise multiple regression analysis: generation and verification of the prediction equation for postoperative ACV

The results of the stepwise multiple regression analysis are shown in Table 3.

**Table 3:** Stepwise multiple regression analysis of factors influencing postoperative ACV in patients with ICL.

Independent Variables	Unstandardized Regression Coefficient	Standardized Partial Regression Coefficient	95% CI	VIF	P
Constant	87.655		−28.268, 203.579		0.137
Preoperative ACV	0.466	0.470	0.271, 0.661	5.359	<0.001
ICL size	−43.036	−0.532	−52.589, −33.483	1.938	<0.001
ACW	40.152	0.602	27.747, 52.558	4.807	<0.001
AOD750	17.003	0.161	2.180, 31.825	2.736	0.025

Values are presented as mean ± standard deviation.

ICL, implantable collamer lens; CI, confidential interval; VIF, variance inflation factor; ACV, Anterior Chamber Volume; ACW, Anterior Chamber Width; AOD, Angle Open Distance

Preoperative ACV, AOD 750, ACW, and ICL size were selected as explanatory variables to predict postoperative ACV (R^2^ = 0.7363). The standardized partial regression coefficients of ACV, ICL size, ACW, and AOD750 were 0.47, −0.532, 0.602, and 0.161, respectively. The prediction equation is as follows:

Postoperative ACV = 87.655 + Preoperative ACV × 0.466 + ICL size × (−43.036) + ACW × 40.152 +AOD750 × 17.003.

**Fig 1:**
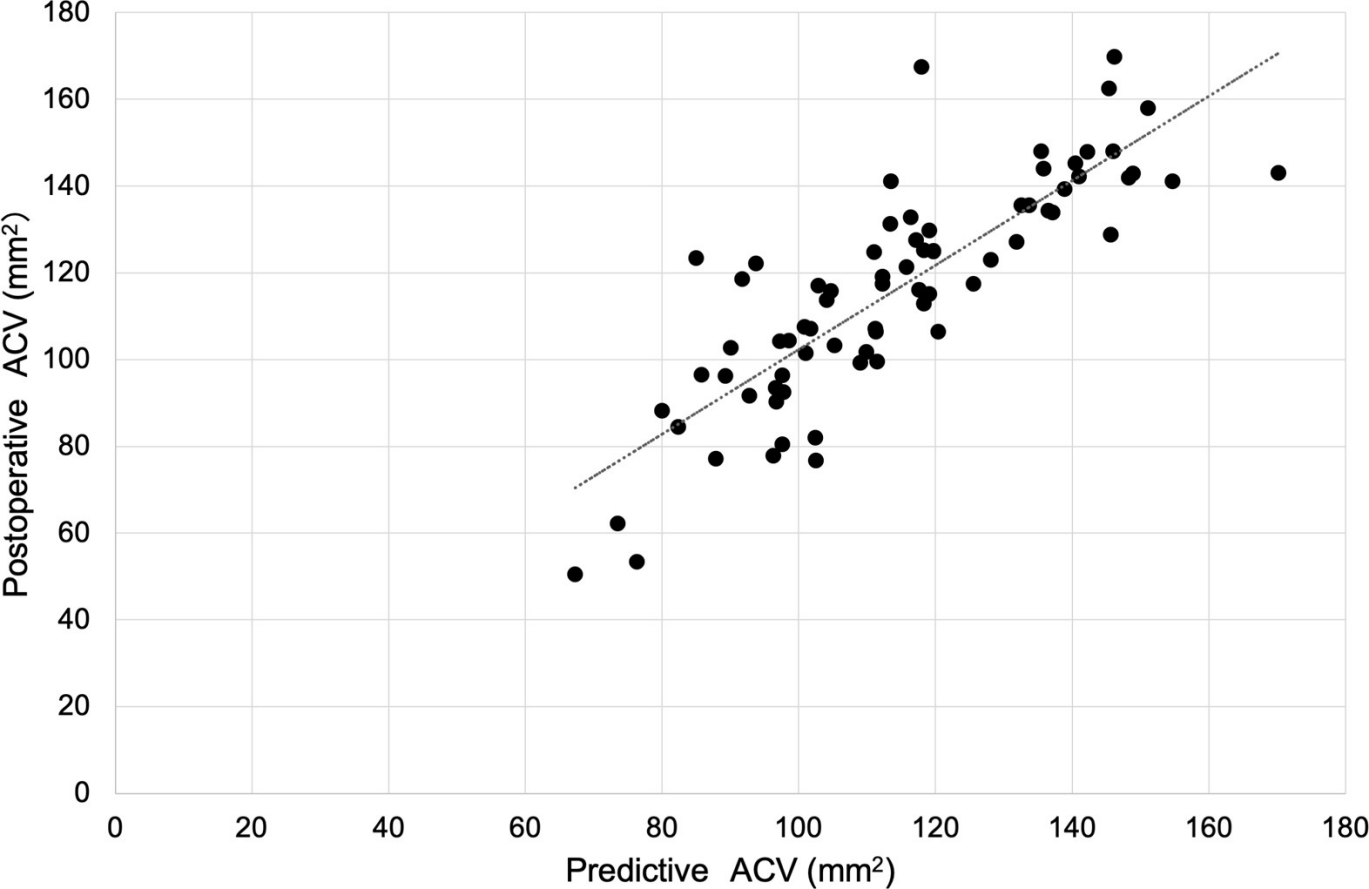
Correlation between the actual and the predictive postoperative anterior chamber volume (ACV). There was a significant correlation between the postoperative and the predicted ACV values (R^2^ = 0.6996, P < 0.001).

**Fig 2:**
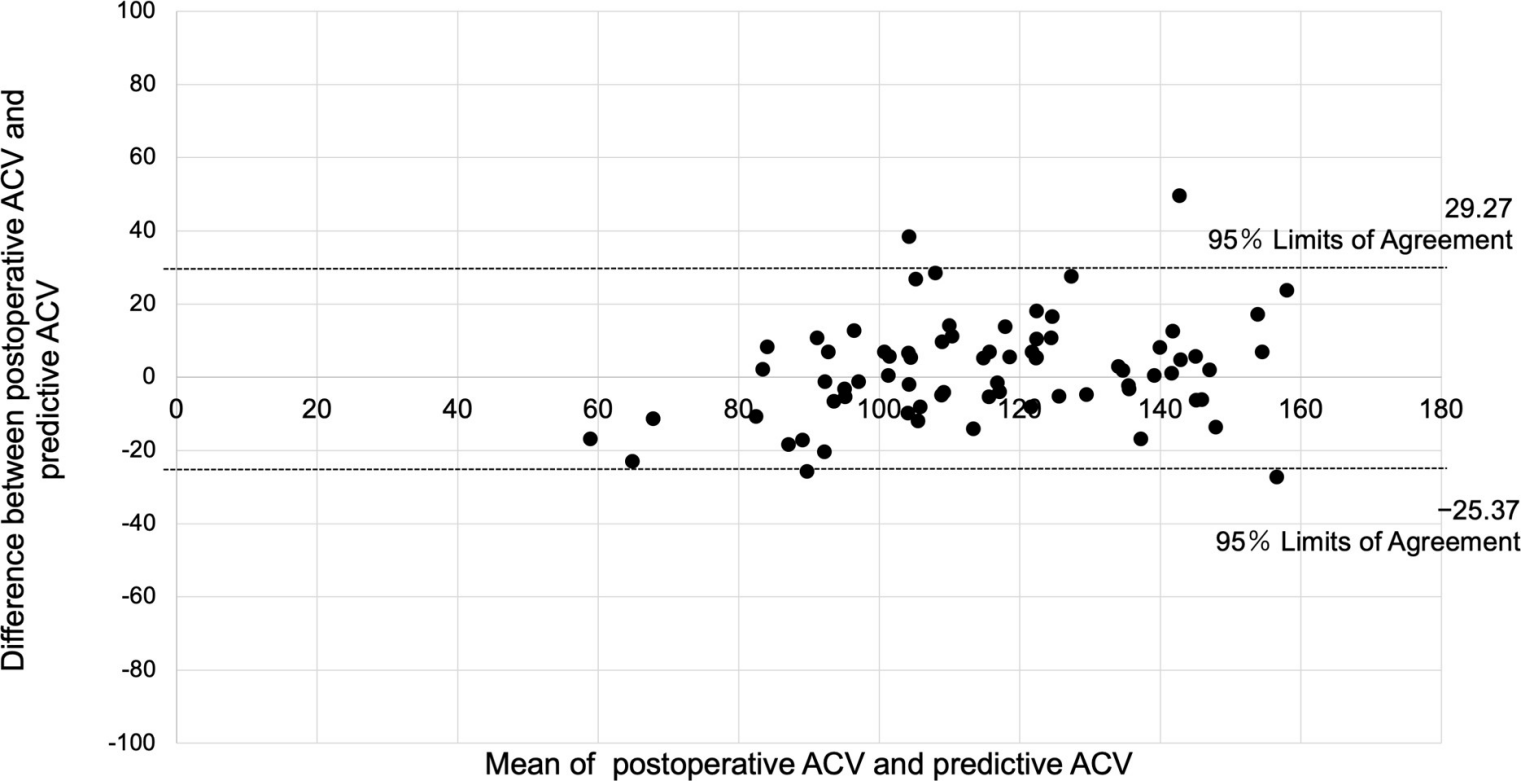
Agreement between the actual postoperative and the predictive anterior chamber volume (ACV). Bland-Altman plot analysis showed no addition and proportional errors between the actual and predicted ACV values.

The predictive postoperative ACV was calculated using the prediction equation in the verification group. Mean predicted (114.2 ± 21.83 mm^3^) and actual postoperative ACVs (116.1 ± 25.41 mm^3^) were not significantly different (P = 0.269), and the mean absolute error was 10.59 ± 9.13 mm^3^. In addition, the correlation between the actual postoperative ACV and predictive ACV was significantly high (adjusted R^2^ = 0.6996, P < 0.0001; Fig 1). In the Bland-Altman plots, there were no addition and proportional errors between actual postoperative and predicted ACV values (Fig 2).

### Case presentation

**Fig 3:**
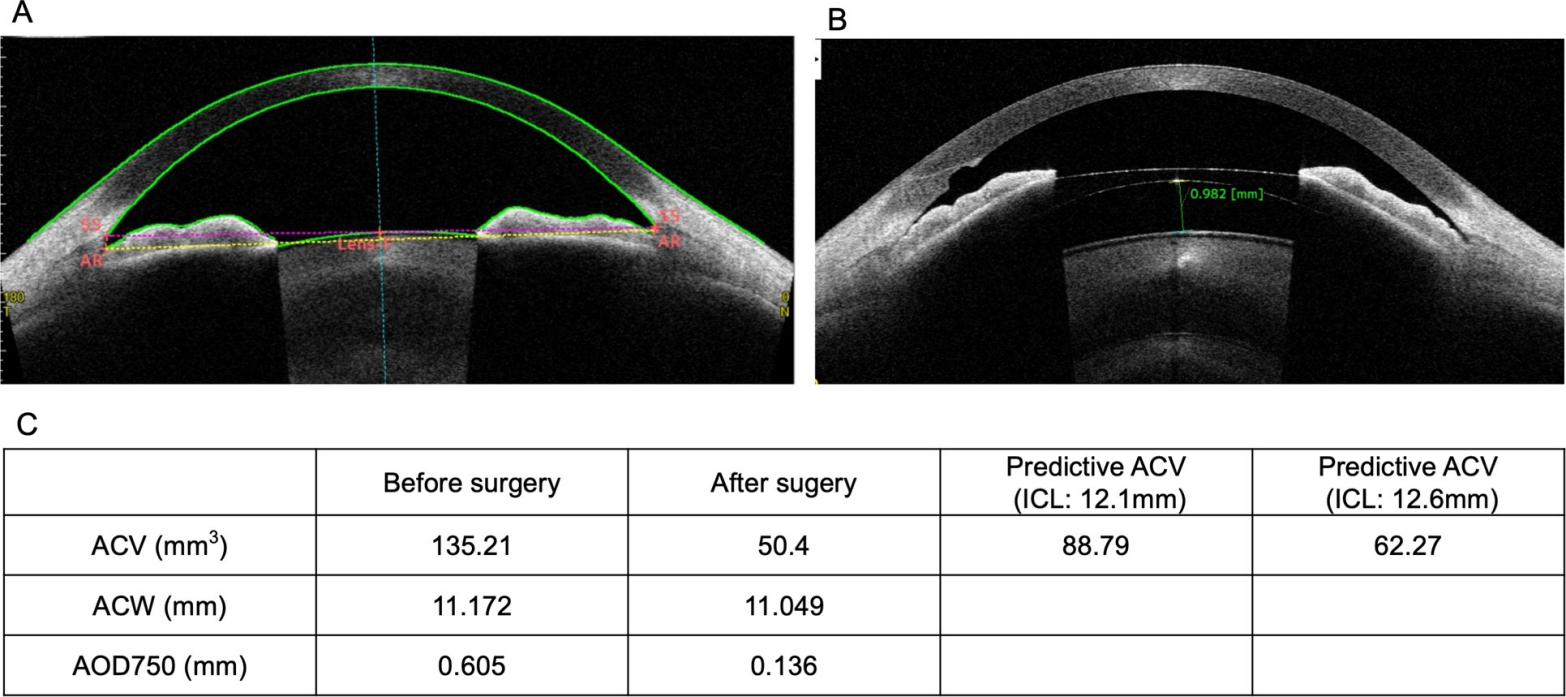
Case presentation showing narrow angle after implantable collamer lens (ICL) implantation. Anterior segment optical computed tomography images at the horizontal meridian before and after ICL implantation are shown (A, before; B, 3 months after surgery). In this case, the ICL size (12.6 mm) was calculated using the vault calculation equation, and a 12.6-mm ICL was implanted. As a result, angle narrowing occurred after surgery. The predictive postoperative ACV value (C) after the 12.6-mm ICL implantation was apparently low, whereas implantation of the 12.1-mm ICL was more suitable according to the postoperative ACV prediction. The generated ACV prediction equation may help determine the appropriate ICL size for such cases.

A representative case (45-year-old woman) is presented in Fig 3. The patient’s preoperative ACV, ACW, AOD750, and corneal diameter were 135.21 mm^3^, 11.172 mm, 0.605 μm, and 11.1 mm, respectively. The ICL size was determined to be 12.6 mm using the nomogram by AS-OCT, according to a previously reported method [4]. At 1 month after surgery, the vault (distance between ICL and anterior crystalline lens) was 1.057 mm high. Slit lamp microscopy revealed that the angle was very narrow. At 3 months after surgery, the ACV, ACW, AOD750, and ICL vault were 50.4 mm^3^, 11.049 mm, 0.136 mm, and 0.982 mm, respectively. The ACV and AOD significantly decreased after the surgery. Moreover, a wide irido-trabecular contact (ITC) area was found (ITC index, 40.2%). However, the IOP was within the normal limit after surgery. The predicted ACVs after implantation of 12.1-mm and 12.6-mm size ICLs were 88.79 mm^3^ and 62.27 mm^3^, respectively. Thus, choosing an ICL of 12.1 mm would have helped avoid the angle narrowing.

## Discussion

The conventional ICL sizing nomogram uses the horizontal corneal diameter and anterior chamber depth. However, this type of nomogram does not consider angle condition, which results in a risk of the angle being narrowed even if the vault is appropriate. Therefore, we tried to create a predictive method that considers the postoperative angle condition.

In the current study, we first analyzed the single correlation of postoperative ACV with AS-OCT parameters and the ICL size. The postoperative ACV showed a strong positive linear correlation with the preoperative ACV and ACD, and a moderate positive correlation with the preoperative ACW, AOD500, AOD750, TISA500, and TISA750. Because angle parameters such as the preoperative AOD and TISA showed moderate correlation with the postoperative ACV, we found that the latter was influenced not only by the anterior chamber structure but also by the angle structure.

According to the stepwise multiple regression analysis using the postoperative ACV as a dependent variable, the ACV, ICL size, ACW, and AOD750 were selected as explanatory variables. The standardized partial regression coefficients of ACV, ICL size, ACW, and AOD750 were 0.47, −0.532, 0.602, and 0.161, respectively. These values indicated that an increase in the ICL size and decrease in the preoperative ACV, ACW, and AOD750 result in a postoperative decrease in the ACV. The coefficient values also indicated that the preoperative ACW had the strongest impact on the postoperative ACV.

The predictive equation for the postoperative ACV included the angle parameter AOD750 in addition to the anterior chamber parameters ACV and ACW, indicating that the calculation formula considered the angle structure in each case. Moreover, the ICL size was selected as an explanatory variable. This seemed to be a reasonable result, considering that the postoperative vault was shown to depend on the ICL size [3]. Furthermore, the regression coefficient of this multiple regression equation was relatively high (0.7363), suggesting that it was possible to preoperatively predict the postoperative ACV at a clinically useful level.

The accuracy of the generated prediction equation was evaluated in the verification group. The absolute error of the ACV was 10.59 ± 9.13 mm^3^, and there was a strong correlation between the actual and predictive ACV values. In addition, there were no addition and proportional errors between the actual and predicted ACV values. These results indicate that the prediction equation is highly accurate and clinically useful to predict the postoperative ACV based on the ICL size.

ICL size was selected as the explanatory variable using multiple regression analysis; however, the single correlation analysis showed no significant correlation with postoperative ACV. This result was consistent with a previous report [13], describing no correlation between ICL size and ACV. It is speculated that ACV may be affected by complex factors, including angle, anterior chamber structure, and ICL size. In addition, only 3 sizes of ICL (12.1mm, 12.6mm, and 13.2mm) were implanted in the current study. The small variation in implanted ICL size may have affected the correlation between ICL size and ACV. Similarly, we found no strong single correlation between ACV and AOD750, although AOD750 was included in the predictive equation.

A previous study used Scheimpflug-based tomography (Pentacam, Oculus, Arlington, WA) for the prediction equation to calculate the postoperative ACV [11]. The authors reported that the postoperative ACV is correlated with the postoperative vault, preoperative ACV, ACD, and horizontal ACA and can also be predicted by these parameters. However, the influence of ICL size and angle parameters, such as AOD, was not examined. Moreover, the prediction of the postoperative ACV was impossible before surgery because the prediction equation included the postoperative ICL vault. Other reports have predicted the trabecular iris angle (TIA) at 1, 3, and 24 months after ICL surgery [14, 15]. Although the prediction equation was calculated from a small number of cases, one report [14] presented an equation that included the ICL size, indicating that the postoperative angle is influenced by the size of the implanted ICL.

The ICL size in the case presented herein was selected using a nomogram considering the postoperative vault height. However, in this case, the postoperative ACV was very low and the ITC area relatively wide after ICL implantation, suggesting an increased risk of angle closure. The predicted ACVs when 12.1-mm and 12.6-mm ICLs were implanted were 88.79 mm^3^ and 62.27 mm^3^, respectively. Because the predicted ACV for the 12.6-mm ICL was very low, implantation of the 12.1-mm ICL may be associated with a reduced risk of postoperative angle narrowing. Thus, it may be necessary to determine the ICL size considering not only the postoperative vault but also the postoperative ACV.

A previous report [6], which detected narrow angle cases with a Shaffer grade ≤ 1 in all four quadrants under gonioscopic observation, showed that the cutoff value of ACV is 113 mm^3^. The average postoperative ACV in our study group was 116.1 ± 25.41 mm^3^, which is close to the previously reported cutoff value. In addition, when examining each case, nearly half of the cases showed postoperative ACV values that were lower than the previously reported cutoff value. We believe that it is important to set a cutoff ACV value to prevent angle closure after ICL surgery.

According to the cross-sectional AS-OCT images, we confirmed that the position and shape of the iris changed after ICL implantation in all cases. Specifically, the ICL lifted the iris, and the overall iris became straight in shape. We speculate that the relatively low ACV value in most eyes after ICL implantation can be explained by the changes in the postoperative position and shape of the iris. In this current study, the iris shape parameter, such as I-curv, was not selected as an explanatory variable of postoperative ACV. Further studies should be performed to analyze the effect of the ICL on the position and shape of the iris.

In our study, AS-OCT measurements were performed under an illuminance level of 150 lux. A previous study reported that the pupil diameter and vault are affected by the illuminance level in the examination room [16]. Because the ACV can also be affected by the illuminance level, the AS-OCT examination should be performed under the same illuminance level when the ACV prediction equation shown in our study is being used.

There are several limitations to our study. First, angle parameters were only measured in the horizontal quadrants. Measurements in the vertical quadrants were difficult in many cases because Asians, including the Japanese, have narrow palpebral fissures. In the future, improvement of the measurement method is necessary to measure parameters in the vertical quadrants for these patients. Second, the average vault was 0.63 ± 0.26 mm in our study. In general, vault sizes ranging between 0.25 mm and 0.75 mm are considered appropriate. In this study, most patients had moderate to large vaults, with only a few having low vaults. Therefore, the accuracy of the ACV prediction equation may be reduced in cases with a small vault. It is necessary to further improve the prediction accuracy by analyzing a larger sample of such cases in the future.

Third, we only followed the patients for 3 months. To the best of our knowledge, no literature has reported long-term changes in ACV after ICL implantation. The ICL vault has been reported to decrease over a long time after ICL implantation [17, 18]. ACV may also change in the long term. Further research is necessary to investigate long-term changes after implantation.

Fourth, although none of the patients showed increased IOP owing to a narrow angle after surgery, some patients showed a wide ITC area postoperatively. Thus, careful long-term follow up of corneal endothelial cell density and IOP is necessary. ITC-area may become an important parameter in the follow up after ICL implantation.

Fifth, in this study, all cases used a myopic model of ICL with a central hole. Our equation may not be useful for no-hole ICLs and hypermetropia ICL models, because those ICLs have different designs and show different aqueous humor dynamics.

Sixth, in the current study, there were no cases that showed angle closure and intraocular pressure rise after surgery. Therefore, we could not calculate the cutoff value of ACV. Future studies that calculate the cutoff value by including many cases at multiple facilities are necessary.

Finally, all patients included in the present study were Japanese. There have been reports of difference in the anterior chamber structures between ethnicities [19, 20]. Further examination is necessary to determine whether the equation is useful for other ethnicities.

In conclusion, our study reveals that the preoperative ACV can be predicted from the ICL size and preoperative AS-OCT parameters, including the ACV, ACW, and AOD750. Considering not only the vault but also the postoperative ACV before surgery, it may be possible to select the proper size of the ICL and reduce the risk of angle narrowing.

## Supporting information

S1 Data (XLSX)

S1 Fig A, Anterior chamber volume (ACV): the volume of the anterior chamber from the corneal endothelium to the lens and iris measured in a zone of 12 mm around the corneal apex (white area). B, Anterior chamber area (ACA): the area of the anterior chamber from the corneal endothelium to the lens and iris (white area). C, Anterior chamber distance (ACD): the distance from the corneal endothelium to the anterior lens capsule (two-way arrow). D, Anterior chamber width (ACW): the distance between scleral spurs (two-way arrow). E, Lens vault (LV): the distance from the line between scleral spurs (SS) to the anterior lens capsule (two-way arrow). F, Vault: the distance from the anterior lens capsule to the posterior ICL (two-way arrow). G, Angle opening distance (AOD): the distance from the posterior corneal endothelium to the anterior iris surface on a line perpendicular to the trabecular meshwork, 250, 500, 750 mm from the scleral spur (two-way arrow). H, Trabecular iris space area (TISA): the area of a square: anteriorly, the AOD at 250, 500, 750 mm from the scleral spur; posteriorly, a line from the scleral spur perpendicular to the plane of the inner scleral wall to the iris; superiorly, the inner corneoscleral wall; and inferiorly, the surface of iris (two-way arrow). I, Angle recess area (ARA): the area from angle recess to AOD at 250, 500, 750 mm from the scleral spur (white area). J, Trabecular iris angle (TIA): the angle of anterior iris and trabecular meshwork (TM) (arrow). K, Iris Curvature (I-Curv): Maximum distance between the posterior iris surface and an imaginary line extending from the iris root to the first point of contact between the iris and the lens (two-way arrow). L, Irido-trabecular contact area (ITC-area): the area of the iris and trabecular meshwork contact (white circle).
(TIF)
