# Supplementary figures and images for: Prediction of anterior chamber volume after implantation of posterior chamber phakic intraocular lens
Source: PLoS One. 2020 Nov 16;15(11):e0242434. doi: 10.1371/journal.pone.0242434 (PMC7668562; doi:10.1371/journal.pone.0242434)

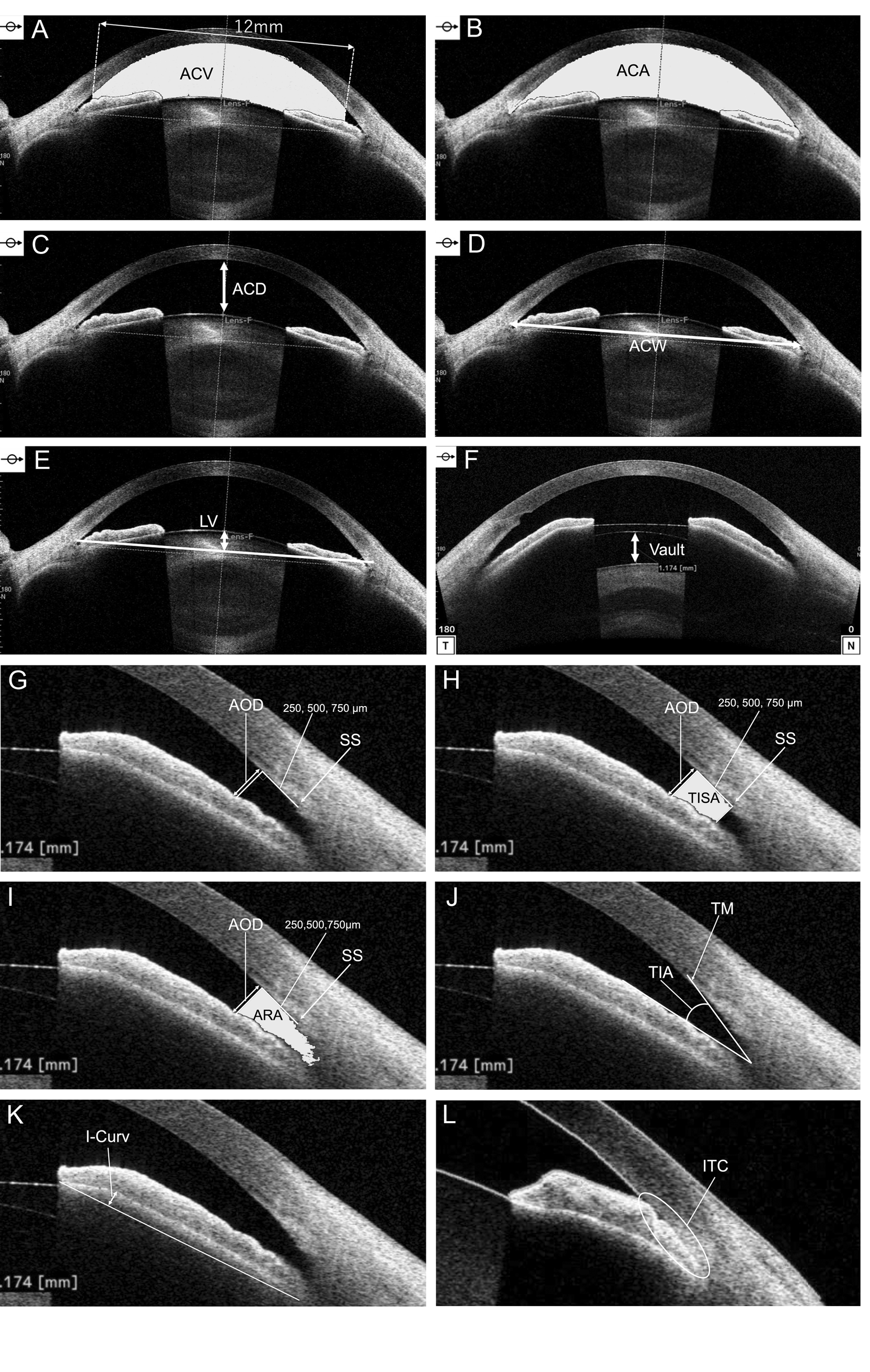

Supplement: S1 Fig — A, Anterior chamber volume (ACV): the volume of the anterior chamber from the corneal endothelium to the lens and iris measured in a zone of 12 mm around the corneal apex (white area). B, Anterior chamber area (ACA): the area of the anterior chamber from the corneal endothelium to the lens and iris (white area). C, Anterior chamber distance (ACD): the distance from the corneal endothelium to the anterior lens capsule (two-way arrow). D, Anterior chamber width (ACW): the distance between scleral spurs (two-way arrow). E, Lens vault (LV): the distance from the line between scleral spurs (SS) to the anterior lens capsule (two-way arrow). F, Vault: the distance from the anterior lens capsule to the posterior ICL (two-way arrow). G, Angle opening distance (AOD): the distance from the posterior corneal endothelium to the anterior iris surface on a line perpendicular to the trabecular meshwork, 250, 500, 750 mm from the scleral spur (two-way arrow). H, Trabecular iris space area (TISA): the area of a square: anteriorly, the AOD at 250, 500, 750 mm from the scleral spur; posteriorly, a line from the scleral spur perpendicular to the plane of the inner scleral wall to the iris; superiorly, the inner corneoscleral wall; and inferiorly, the surface of iris (two-way arrow). I, Angle recess area (ARA): the area from angle recess to AOD at 250, 500, 750 mm from the scleral spur (white area). J, Trabecular iris angle (TIA): the angle of anterior iris and trabecular meshwork (TM) (arrow). K, Iris Curvature (I-Curv): Maximum distance between the posterior iris surface and an imaginary line extending from the iris root to the first point of contact between the iris and the lens (two-way arrow). L, Irido-trabecular contact area (ITC-area): the area of the iris and trabecular meshwork contact (white circle). (TIF) [file pone.0242434.s002.tif]
